# Evaluation of Sex Distribution of Industry Payments Among Radiation Oncologists

**DOI:** 10.1001/jamanetworkopen.2018.7377

**Published:** 2019-01-25

**Authors:** Julius K. Weng, Luca F. Valle, Gina E. Nam, Fang-I Chu, Michael L. Steinberg, Ann C. Raldow

**Affiliations:** 1 Department of Radiation Oncology, David Geffen School of Medicine at UCLA, Los Angeles, California; 2 Department of Epidemiology, Fielding School of Public Health, University of California, Los Angeles

## Abstract

**Importance:**

Industry relationships are an important measure of professional advancement; however, the association between physician sex and industry payments in radiation oncology has not been described.

**Objective:**

To update the trends in the sex distribution of industry payments in radiation oncology.

**Design, Setting, and Participants:**

This retrospective cross-sectional study was conducted between July 1, 2018, and August 31, 2018. It used the publicly available Centers for Medicare & Medicaid Services (CMS) Open Payments program and CMS Physician and Other Supplier Public Use File databases to obtain 2016 industry payment data for US radiation oncologists who reported receiving industry funding in that year (n = 3052). Total monetary value, number of payments, and median payment amounts were determined for each sex in the following categories: research, consulting, honoraria, industry grants, royalty or license, and services other than consulting.

**Main Outcomes and Measures:**

Industry payment amounts among 3052 radiation oncologists who reported receiving payments in 2016; association of median payment with the types of payment by sex.

**Results:**

Of the total 4483 radiation oncologists who practiced in 2016, 1164 (25.9%) were female and 3319 (74.0%) were male. Industry payments were distributed among 3052 radiation oncologists (68.1%), of whom 715 (23.4%) were female and 2337 (76.6%) were male. The proportion of female radiation oncologists who received at least 1 industry payment was 61.4% (715 of 1164), whereas the proportion of their male counterparts was 70.4% (2337 of 3319). Across all payment types, female radiation oncologists received a smaller percentage of total industry funding than the percentage of female physicians represented in each category. The median payment value was smaller for female radiation oncologists in consulting (–$1000; 95% CI, −$1966.67 to $100.63; *P* = .005) and honoraria (–$500; 95% CI, −$1071.43 to $0; *P* = .007). This trend was also observed in research payments, but was not statistically significant (–$135.02; 95% CI, −$476.93 to $6.88; *P* = .08). Of the $1 347 509 royalty or license payments made to 72 physicians, none was for female radiation oncologists.

**Conclusions and Relevance:**

Distribution of industry payments appears to show sex disparity in industry relationships among radiation oncologists; this observation warrants further investigation to determine the underlying reasons and provide avenues for increased parity.

## Key Points

**Question:**

What is the sex distribution of industry payments in radiation oncology?

**Findings:**

In this cross-sectional study involving 4483 radiation oncologists, the proportion of radiation oncologists who received at least 1 industry payment in 2016 was substantially lower among female physicians (61.4%) than their male counterparts (70.4%). Across all payment types, female radiation oncologists received a smaller percentage of the total industry funding than their corresponding representation in these categories.

**Meaning:**

Distribution of corporate payments appears to show sex disparity in industry relationships among radiation oncologists; further investigation is needed to increase parity.

## Teaser

This cross-sectional study uses the 2016 self-reported industry payment data from the Centers for Medicare & Medicaid Services (CMS) Open Payments and CMS Physician and Other Supplier Public Use File databases to investigate trends in funding distribution by sex among radiation oncologists.

## Introduction

Sex inequity has been extensively described in academic medicine,^1,2,3^ including radiation oncology.^4,5^ Although the number of female medical school matriculants has steadily increased and now surpasses the number of their male counterparts,^6^ female radiation oncologists continue to represent less than one-third of physicians.^7^ This discrepancy has remained stagnant,^8^ despite national recognition^9,10,11,12^ and the substantial progress made in narrowing the gap within medical oncology.^13^ The disparity in sex representation is even more pronounced in positions of seniority within radiation oncology, with disproportionately fewer female chairpersons, American Society for Radiation Oncology board members and presidents, and gold medal recipients,^4,14,15^ compared with their male counterparts.

In addition to academic promotion and societal recognition, industry partnership may also serve as a measure of sex disparity. Industry relationships, although controversial,^16,17,18^ provide substantial career advantages in the form of research funding and access to novel therapeutics and may indicate industry recognition as a key opinion leader.^19^ The introduction of the Centers for Medicare & Medicaid Services (CMS) Open Payments program^20^ as part of the Physician Payments Sunshine Act has permitted the increased scrutiny of physicians’ industry relationships. Previous work that used this publicly available database has demonstrated sex inequity in industry payments across multiple specialties.^21,22,23,24^ More than 50% of radiation oncologists received industry payments in 2014,^25,26^ but the sex distribution of these payments has not been characterized. This cross-sectional study that used the 2016 CMS Open Payments database sought to update the trends in radiation oncology industry payments and specifically evaluate these payments for sex inequalities.

## Methods

### Data Sources

This retrospective cross-sectional study was conducted between July 1, 2018, and August 31, 2018, and used the publicly available CMS Open Payments program^20^ and CMS Physician and Other Supplier Public Use File^27^ databases. Payment data were obtained for all radiation oncologists in the United States who were reported in the 2016 CMS Open Payments database to have received industry payments. In 2016, the CMS Open Payments program mandated industry reporting of individual payments greater than $10.22 or total payments greater than $102.19 in a calendar year. The total number of radiation oncologists who participated in CMS during 2016 was obtained from the CMS Physician and Other Supplier Public Use File database.^27^ As all data were publicly available, this study qualified for UCLA Institutional Review Board exemption and did not require informed consent. This study followed the Strengthening the Reporting of Observational Studies in Epidemiology (STROBE) reporting guideline.

### Payments

The types of industry payments are categorized by CMS as research, ownership, and general payments. General payments are further categorized into 12 payment types as consulting fees, services other than consulting (ie, serving as faculty or as a speaker), honoraria, grants in support of a specific cause or activity, royalty or license, charitable contributions, education, food and beverage, travel and lodging, gift, current or prospective ownership or investment interest, and entertainment. Definitions of these categories can be found on the CMS website.^28^ Similar to previously reported analyses,^22,24^ the present study focused on categories of payments considered to represent substantial industry-physician relationship: research, consulting, services other than consulting, honoraria, grants related to industry ownership, and royalty or license. The remaining payments, including charitable contributions, education, food and beverage, travel and lodging, entertainment, and gift, were considered less substantive ties and were examined in an exploratory analysis.

### Sex

Sex was initially ascertained by cross-referencing the first names of radiation oncologists with the 2016 CMS Physician and Other Supplier Public Use File database.^27^ If sex was inconclusive, it was clarified through internet search of individual physician names. Similar methods have been previously described in an analysis of other specialties.^22^

### Statistical Analysis

Descriptive statistics of payments were calculated by sex for each industry payment type. The median differences and 95% CIs were estimated via quantile regression models. A 2-tailed Wilcoxon rank sum test was used to compare the medians for each sex. Comparisons of median payment amounts were not performed if the sample size was fewer than or equal to 2 for female or male. For all statistical tests, the significance level was set as a 2-sided *P* = .05. All analyses were carried out in R statistical software, version 3.5.0 (R Foundation for Statistical Computing).^29^

## Results

Among a total of 4483 radiation oncologists practicing in 2016, 1164 (25.9%) were female and 3319 (74.0%) were male (Table 1). Industry payments were distributed among 3052 radiation oncologists (68.1%), of whom 715 (23.4%) were female and 2337 (76.6%) were male. The proportion of female radiation oncologists who received at least 1 industry payment was 61.4% (715 of 1164), whereas the proportion of their male counterparts was 70.4% (2337 of 3319).

**Table 1.** Overall Radiation Oncologist Ties to Industry in 2016

Sex	No. of Radiation Oncologists With Open Payments Transaction (%)^a^	Total No. (%) of Radiation Oncologists
All	3052	4483
Female	715 (23.4)	1164 (25.9)
Male	2337 (76.6)	3319 (74.0)

^a^ Payments reported to the Centers for Medicare & Medicaid Services Open Payments program.

Payments in research, consulting, honoraria, industry grants, royalty or license, and services other than consulting, education, entertainment, food and beverage, and travel and lodging are reported by category in Table 2. Charitable contributions, current or prospective ownership, gift, and general grants are not reported because of the few number of transactions (<10). The category *all* in Table 2 is the summation of the 14 payment categories. Individuals can be assigned to more than 1 type of payments, and not all payment types are listed in Table 2. Therefore, the total numbers of females and males in Table 2 do not reflect the total numbers of females and males in Table 1. Of the 6 listed categories of industry payments, consulting was the most common payment type (n = 369) and had the greatest total monetary value ($2 745 242) among these 6 categories. In contrast, industry grants were awarded to the fewest number of physicians (n = 21), but it was the category with the second largest total monetary value ($2 467 906). Research payments (n = 29) represented the smallest total monetary value ($208 005).

**Table 2.** Monetary Value of Industry Payments to Radiation Oncologists in 2016

Category	No. of Payments (%)	US $			Median Difference Between the Sexes (95% CI), US $	*P* Value for Median
		Total Payments (%)	Median Payments (IQR)	Maximum Payments		
All^a^						
All	25 700	9 744 992.8	16.85 (26.21)		−0.88 (−1.20 to −0.56)	
Female	4581 (17.82)	479 561.4 (4.9)	16.14 (11.44)	50 000.00	NA	<.001
Male	21 119 (82.18)	9 265 431.4 (95.1)	17.02 (31.99)	83 694.30	NA	
Research						
All	29	208 005.10	45.38 (450.70)		−135.02 (−476.93 to 6.88)	
Female	6 (20.7)	349.24 (0.2)	38.74 (12.25)	177.20	NA	.08
Male	23 (79.3)	207 655.90 (99.8)	177.20 (963.01)	191 972.20	NA	
Consulting						
All	369	2 745 242.00	3000.00 (5050.00)		−1000 (−1966.67 to 100.63)	
Female	30 (8.1)	65 951.00 (2.4)	2000.00 (2537.50)	6000.00	NA	.005
Male	339 (91.9)	2 679 291.00 (97.6)	3000.00 (5239.50)	198 082.00	NA	
Honoraria						
All	228	466 154.00	2000.00 (1300.00)		−500 (−1071.43 to 0)	
Female	29 (12.7)	42 616.16 (9.1)	1500.00 (1250.00)	4000.00	NA	.007
Male	199 (87.3)	423 537.8 (90.9)	2000.00 (1009.59)	13 163.00	NA	
Industry grants						
All	21	2 467 906.00	50 160.00 (59 500.00)		−2646.14 (−52 000 to 7500)	
Female	2 (9.5)	99 400.00 (4.0)	49 700.00 (300.00)	50 000.00	NA	NA
Male	19 (90.5)	2 368 506 (96.0)	52 646.14 (82 600.00)	836 394.30	NA	
Royalty or license						
All	72	1 347 509.00	1272.75 (11 896.24)		−1272.75 (NA)	
Female	0	NA	NA	NA	NA	NA
Male	72 (100.0)	1 347 509.00 (100.0)	1272.75 (11 896.24)	214 785.50	NA	
Other services						
All	340	949 672.40	2200.00 (2681.75)		−900 (−1550 to 1800)	
Female	17 (5.0)	38 694.84 (4.1)	1300.00 (3125.00)	6500.00	NA	.30
Male	323 (95.0)	910 977.50 (95.9)	2200.00 (2548.75)	35 579.00	NA	
Education						
All	211	18 628.19	15.91 (32.82)		−2.18 (−6.14 to 3.84)	
Female	35 (16.6)	1177.19 (6.3)	13.98 (14.03)	150.00	NA	.42
Male	176 (83.4)	17 451.00 (93.7)	16.58 (35.59)	7280.00	NA	
Entertainment						
All	24	887.91	23.6 (30.74)		−11.26 (−30.74 to 23.86)	
Female	3 (12.5)	66.05 (7.4)	12.53 (16.51)	43.27	NA	.38
Male	21 (87.5)	821.86 (92.6)	23.79 (30.74)	153.75	NA	
Food and beverage						
All	22 734	688 169.70	15.84 (10.34)		−0.12 (−0.42 to 0.09)	
Female	4293 (18.9)	126 908.2 (18.4)	15.75 (9.36)	512.72	NA	.91
Male	18 441 (81.1)	561 261.5 (81.6)	15.87 (10.58)	2306.00	NA	
Travel and lodging						
All	1655	843 809.9	200.0 (380.16)		19.43 (−36.07 to 83.66)	
Female	163 (9.8)	104 088.2 (12.3)	216.31 (368.03)	7935.10	NA	.15
Male	1492 (90.2)	739 721.7 (87.7)	196.84 (383.20)	12 497.16	NA	

Abbreviations: IQR, interquartile range; NA, not applicable.

^a^ *All* is the sum of the 14 payment categories. Individuals can be assigned to more than 1 type of payment, and not all payment types are listed. Therefore, the total numbers of female and male radiation oncologists do not reflect the total numbers in Table 1.

Across all payment types, female radiation oncologists received a smaller percentage of total industry funding than their corresponding representation in these categories. In considering payment amounts by individual (Figure), the median payment value was smaller for female radiation oncologists in consulting (–$1000; 95% CI, −$1966.67 to $100.63; *P* = .005) and honoraria (–$500; 95% CI, −$1071.43 to $0; *P* = .007). This trend was also observed in research payments, but was not statistically significant (–$135.02; 95% CI, −$476.93 to $6.88; *P* = .08). Female representation was the greatest in research (n = 6 [20.7%]), but female radiation oncologists received only 0.2% of total research payments. No statistically significant difference in median payments was found for services other than consulting (−$900; 95% CI, −$1550 to $1800; *P* = .30), education (−$2.18; 95% CI, −$6.14 to $3.84; *P* = .42), entertainment (−$11.26; 95% CI, −$30.74 to $23.86; *P* = .38), food and beverage (−$0.12; 95% CI, −$0.42 to $0.09; *P* = .91), or travel and lodging ($19.43; 95% CI, −$36.07 to $83.66; *P* = .15). Of note, the differences in median payments could not be assessed for industry grants and royalty or license because of the limited number of female radiation oncologists observed for these 2 categories. Of the $1 347 509 royalty or license payments made to 72 physicians, none was for female radiation oncologists.

**Figure.**
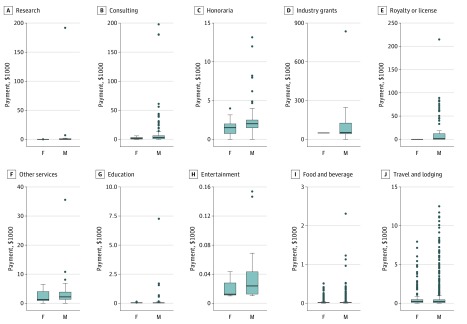
Distribution of 2016 Industry Payments by Payment Type and Physician Sex Bolded line represents median. Box ends represent first and third quartiles. Whisker ends represent minimum and maximum values. Individual points represent outliers. F indicates female radiation oncologists; M, male radiation oncologists.

## Discussion

In this study of 4483 radiation oncologists, 68.1% received at least 1 industry payment in 2016, compared with prior estimates of approximately 52% to 58% in 2014.^25,26^ This surge of industry relationships may not have benefitted female and male radiation oncologists equally. Despite 25.9% of radiation oncologists being female, they represented less than 15% of physicians in each assessed category, with the exception of research, in which 20.7% female radiation oncologists were represented. However, these physicians received only 0.2% of total research payments. This trend of receiving lower median payments and representing a smaller-than-expected percentage of the total dollar amount of payments was consistent across all categories.

It is well established that women in academic medicine face obstacles in achieving parity in many metrics of career success, including salary,^30^ research funding,^31^ and academic promotion.^1^ Industry relationships, controversies notwithstanding, are an important measure of professional advancement and financial support. Our findings of fewer number of payments and payments of lower monetary value to female physicians are consistent with reports in other historically male-dominated specialties, such as ophthalmology,^22^ otolaryngology,^23^ and general surgery,^24^ as well as with nationwide studies of older CMS Open Payments program data.^32,33^ For instance, the dearth of royalty or licensing payments is by no means a finding specific to radiation oncology. A national study determined that male surgeons were approximately 43 times, primary care physicians 9 times, specialists 4 times, and interventionists 8 times more likely to receive royalty payments than their female counterparts.^33^ Although not assessed in this study, industry payments appear to be more sex biased at institutions with higher reputation^21^ and become progressively disparate with career advancement from assistant to full professorship.

In the context of the aforementioned disparities, no difference in median industry research payments was found between male and female radiation oncologists. This finding is in contrast to several studies reporting unequal sex distribution of R01 awards,^34^ overall National Institutes of Health funding in radiation oncology^15^ and surgical subspecialties,^31,35^ as well as federal and nonfederal funding at a single institution.^36^ However, on further analyses, these sex disparities in research funding were partially or completely ameliorated after adjusting for academic rank. The observed sex parity in research payments may represent an industry selection bias for uniformly senior faculty. This bias may stem from an industry desire to recruit influential key opinion leaders. Alternatively, the observed parity may be the result of being statistically underpowered because of the small cohort—less than 1% of individuals with industry relationships received research payments in 2016.

This study only permits speculation on the reason for the observed sex disparity. The inequality may stem from industry preference for established senior faculty, which is known to disproportionately comprise male physicians in radiation oncology.^15^ The industry may also favor faculty who demonstrate expertise through academic productivity. Receiving industry funding appears to be correlated with increases in publication indexes,^37^ known to be lower among female junior radiation oncologists compared with their male colleagues.^15^ Expertise may also be measured by holding patents, another metric that is substantially lower among female physicians.^38^ These suppositions imply that sex disparities in multiple arenas may beget sex disparities in industry payments. Like a self-fulfilling prophecy, inequity in one context may set a precedent for inequity in another, both of which independently and cumulatively contribute to greater disparity.

All of the aforementioned disparities may be downstream consequences of sex gaps experienced early in a female physician’s career. For instance, mentorship and sponsorship are both crucial to developing and negotiating industry relationships. Surveys of radiation oncology residents reveal that female residents are more likely than male residents to have difficulty finding a mentor.^39^ Furthermore, female radiation oncology residents with families report taking a greater share of child care responsibilities, compared with their fellow male residents.^40^ These findings are consistent with a study of young female physician researchers with children, who reported spending more time on domestic duties as compared with their male counterparts.^41^ These additional non–career-oriented responsibilities of female physicians could affect their desire and time availability to pursue industry relationships.

Sex differences in socialization could also be a factor in the decision to seek industry relationships. Women have historically been associated with communal qualities, whereas men have been characterized by agentic traits.^42^ These stereotypes are theorized to contribute to sex variability in ethical reasoning.^42^ As a generalization, women are less likely to pursue business opportunities involving perceived ethical compromises and are more likely to identify ethical concerns as compared with men.^42,43^ In contrast, men appear to be more egocentric in business negotiations and have a greater willingness to accept ethical uncertainty.^42,43^ In the context of industry relationship, female physicians may be more likely to avoid ethically ambiguous partnership that may compromise appropriate clinical decision-making or add unnecessary cost to the health care system.

The sex disparity in industry payments may represent female physicians choosing not to pursue industry relationships. Of greater concern is the possibility that this observed disparity may be a proxy for the systemic inequalities that female physicians face in radiation oncology. A first step in clarifying the origin of this gap could be incorporating industry-related questions into a workforce survey. If the observed inequality is then suspected to be a true disparity, the most relevant metric may be female physicians receiving a percentage of total industry funding that corresponds to their representation in the field.

### Limitations

This study has several limitations. First, our analyses relied on the CMS Open Payments program database, which, like all databases, is reliant on self-reports and is subject to reporting bias and missing data. However, 2016 marks the fourth year of the Open Payments program, which continues to be improved, implements initiatives to increase industry compliance, and encourages physician review of their own data. Second, the total number of radiation oncologists in the United States in 2016 cannot be determined. The number of physicians in the 2016 CMS data set was determined by National Provider Identifiers and in Open Payments data set by physician profile IDs. To verify whether the 2016 CMS data set contained all radiation oncologists in the Open Payments data set, we compared 2 data sets using the exact match of physicians’ full names. Of the 3052 radiation oncologists included in this analysis, 408 were not found in the 2016 CMS data set. This may indicate that the 2016 CMS data set does not represent a total number of radiation oncologists in the United States or that 1 of the 2 databases includes radiation oncologists more accurately to reflect their activities during 2016. Third, this study is limited by the unavailable demographic information in the Open Payments program database. This missing information restricted our investigation into sex disparity by factors such as physician age, years of practice, subspecialties in radiation oncology, and geographic region of practice.

## Conclusions

The Physician Payments Sunshine Act, although originally intended to provide insight into problematic conflicts of interest, has shed light on sex inequality in industry relationships. However, the identification and surveillance of sex inequality may only be a step toward parity. The path toward mitigating inequity likely must be multifaceted, and numerous strategies for addressing these barriers have recently emerged.^44^ It is incumbent on all of us—regardless of sex—to advocate for and work toward equality.
